# Responsiveness of Inhaled Epoprostenol in Respiratory Failure due to COVID-19

**DOI:** 10.1177/0885066620976525

**Published:** 2020-11-25

**Authors:** Rajiv Sonti, C. William Pike, Nathan Cobb

**Affiliations:** 1 Division of Pulmonary, Critical Care and Sleep Medicine, 12230 Georgetown University Medical Center, Washington, DC, USA; 2 12230 Georgetown University School of Medicine, Washington, DC, USA

**Keywords:** inhaled epoprostenol, ARDS, COVID-19

## Abstract

**Background:**

Inhaled pulmonary vasodilators are used as adjunctive therapies for the treatment of refractory hypoxemia. Available evidence suggest they improve oxygenation in a subset of patients without changing long-term trajectory. Given the differences in respiratory failure due to COVID-19 and “traditional” ARDS, we sought to identify their physiologic impact.

**Methods:**

This is a retrospective observational study of patients mechanically ventilated for COVID-19, from the ICUs of 2 tertiary care centers, who received inhaled epoprostenol (iEpo) for the management of hypoxemia. The primary outcome is change in PaO_2_/FiO_2_. Additionally, we measured several patient level features to predict iEpo responsiveness (or lack thereof).

**Results:**

Eighty patients with laboratory confirmed SARS-CoV2 received iEpo while mechanically ventilated and had PaO_2_/FiO_2_ measured before and after. The median PaO_2_/FiO_2_ prior to receiving iEpo was 92 mmHg and interquartile range (74 – 122). The median change in PaO_2_/FiO_2_ was 9 mmHg (-9 – 37) corresponding to a 10% improvement (-8 – 41). Fifty-percent (40 / 80) met our a priori definition of a clinically significant improvement in PaO_2_/FiO_2_ (increase in 10% from the baseline value). Prone position and lower PaO_2_/FiO_2_ when iEpo was started predicted a more robust response, which held after multivariate adjustment. For proned individuals, improvement in PaO_2_/FiO_2_ was 14 mmHg (-6 to 45) vs. 3 mmHg (-11 – 20), p = 0.04 for supine individuals; for those with severe ARDS (PaO_2_/FiO_2_ < 100, n = 49) the median improvement was 16 mmHg (-2 – 46).

**Conclusion:**

Fifty percent of patients have a clinically significant improvement in PaO_2_/FiO_2_ after the initiation of iEpo. This suggests it is worth trying as a rescue therapy; although generally the benefit was modest with a wide variability. Those who were prone and had lower PaO_2_/FiO_2_ were more likely to respond.

## Background

Inhaled pulmonary vasodilators, nitric oxide (iNO) and epoprostenol (iEpo), are used for refractory hypoxemia of the acute respiratory distress syndrome (ARDS). There is attractive physiologic rationale: the vasodilators are delivered to the diseased lung from the airways, leading to increased perfusion of well-ventilated lung units thereby improving shunt fraction.^1^ Available evidence suggests these therapies do not improve mortality,^2,3^ but do improve oxygenation in a subset of patients.^4-7^ Inhaled epoprostenol is thought to be equally efficacious but is less expensive than iNO.^8,9^

High quality data demonstrating benefits of iEpo other than a short-term improvement in oxygenation are limited; therefore, a recent Cochrane review did not recommend for or against its use, instead suggesting the need for additional studies.^10^ In practice, patients are often trialed on iEpo alongside other proven therapies, such as lung protective ventilation,^11^ prone positioning,^12^ conservative fluid management,^13^ and the use of neuromuscular blockade^14,15^ in select cases. In a recent landmark multicenter trial focused on the efficacy of extracorporeal membrane oxygenation (ECMO), 83% of patients who did not receive ECMO (and 60% who did) were trialed on iNO or iEpo.^16^

Despite limitations of inhaled pulmonary vasodilators in ARDS in general, there is some reason to believe they may be specifically beneficial in respiratory failure due to COVID-19. Though the precise pathophysiology remains unclear, it appears that at least a subset of those with COVID-19 have hypoxemia out of proportion to imaging findings with preserved lung mechanics,^17^ a pattern characteristic of pulmonary vascular disease. In addition, autopsy studies (limited by small numbers) show vascular changes along with the presence of microthrombi.^18,19^ Transthoracic echocardiography studies demonstrate a tendency for increased right ventricular strain, which has been correlated with prognosis.^20,21^ Even outside the lung, there is emerging evidence that SARS-CoV-2 is a diffusely vasculotropic virus.^22^ In addition to being vasodilatory, prostaglandins may have pleotropic anti-inflammatory and anti-platelet effects.^1^

Professional societies currently recommend against the routine use of inhaled vasodilators in COVID-19 respiratory failure, based on weak evidence.^23,24^ Therefore, we sought to identify the physiologic response (as measured by PaO_2_/FiO_2_) to iEpo in patients with severe respiratory failure due to COVID-19. We additionally attempted to determine if there were patient level factors that predicted responsiveness (or lack thereof) given the emphasis on deliberate resource utilization with scarcities brought about by the pandemic.

## Methods

This is a retrospective study involving 2 tertiary care centers in a single heath system in Washington DC: Medstar Georgetown University Hospital and Medstar Washington Hospital Center. All consecutive patients from 3/1/2020 to 5/22/2020 were included if diagnosed with laboratory confirmed SARS-CoV2, were mechanically ventilated for hypoxemic respiratory failure and received inhaled epoprostenol (iEpo). The hospitals share a single set of clinical guidelines for the care of COVID patients; however, this does not address iEpo. Therefore, it was used at the treating clinician’s discretion. Dosing was started at 50 nanograms per kilogram of ideal body weight per minute, delivered via a syringe through an infusion pump attached to the inspiratory limb of the ventilator tubing. The primary outcome is change in the ratio of arterial oxygen pressure to the fraction of inhaled oxygen delivered by the ventilator, PaO_2_/FiO_2_, measured before and after the initiation of iEpo.

In order to isolate the specific impact of iEpo we excluded patients if there were changes in the ventilator mode, positive end-expiratory pressure (PEEP), FiO_2_, patient position (from prone to supine or vice versa) or initiation / removal of neuromuscular blockade between PaO_2_/FiO_2_ measurements. Up to 24 hours were allowed between arterial blood gas measurements and the iEpo initiation (before and after) if these criteria were met. Changes in tidal volume (Vt) and respiratory rate (RR) were permitted. We defined a clinically significant improvement in PaO_2_/FiO_2_ as a 10% increase (“iEpo responders”), based on our perception of a reasonable, clinically useful change and a definition that has been previously used.^25^ Patients on extracorporeal membrane oxygenation (ECMO) were excluded.

In order to determine if there were patient features that predicted improvement, we recorded variables potentially related to iEpo responsiveness based on biologic plausibility and prior data^4,7^; demographics, past medical history, severity of illness as measured by the sequential organ failure assessment (SOFA) score^26^ ventilator parameters (mode, FiO_2_, PEEP, Vt, RR, plateau pressure, driving pressure [plateau pressure – PEEP], static compliance [Vt / driving pressure]), prone position, net fluid balance, use of specific medications (neuromuscular blockade, therapeutic anticoagulation, steroids) and laboratory data (C-reactive protein, D-dimer, Il-6). Prone position was defined as the patient being physically proned at the time of iEpo administration. Neuromuscular blockade was defined as a continuous infusion of cisatricurium or having received vecuronium within 2 hours of iEpo initiation. Steroid use was defined as the administration of at least 40 mg of methylprednisolone (or equivalent) within 24 hours. Anticoagulation referred to therapeutic dosing of heparin or enoxaparin (at the time of iEpo administration). Laboratory data were values most proximate to iEpo initiation.

Data was obtained directly from the health system’s clinical data warehouse and supplemented with direct review of patient records when needed. Summary statistics describe the frequency of each categorical variable and either mean (for normally distributed) or median (for non-normally distributed) of continuous variables. In a comparison between responders and non-responders to iEpo, continuous data were compared via the student t-test and Wilcoxon rank sum test for normally and non-normally distributed data respectively. Categorical data were analyzed with a Chi-Square test or Fisher’s Exact test as appropriate. We included variables with statistically significant univariate associations along with others that were clinically relevant as candidates in regression models with change in PaO_2_/FiO_2_ as the dependent variable of interest, developed in a stepwise fashion with a stopping rule based on minimum Bayesian Information Criterion (BIC). Data extraction, cleaning and pre-processing were performed in R, and analysis using JMP 15 Pro (Cary, NC). This study was approved by the Institutional Review Board of Georgetown University.

## Results

### Patient Characteristics

During the study period, 80 patients invasively mechanically ventilated (iMV) for respiratory failure due to COVID-19 received iEpo and met inclusion criteria. Demographic features and past medical history are described in Table 1. The median age was 59, 59% were male, 56% Black; the majority had at least one co-morbidity including diabetes mellitus (40%) and morbid obesity (18%). The time between intubation and the initiation of iEpo was highly variable with a median of 17 hours and interquartile range (8 – 74). The total time on iMV was a median of 13 days (7 – 21) and 48 patients (60%) died while hospitalized (the remainder were discharged alive).

**Table 1.** Patient Characteristics.

	Total (n = 80)	iEpo responder (n = 40)	iEpo non-responder (n = 40)	p-value
**Age**, median (IQR)	59 (48 – 67)	60 (49 – 69)	59 (46 – 66)	0.44
**Male**, n (%)	47 (59)	26 (65)	21 (53)	0.25
**BMI**, median (IQR)	30 (27 – 38)	31 (28 – 39)	30 (28 – 37)	0.42
**Morbidly obese (BMI > 40)**, n (%)	14 (18)	7 (18)	7 (18)	0.99
**Race**, n (%)				0.94
** Black or African American**	45 (56)	22 (55)	23 (58)	
** White**	7 (9)	4 (10)	3 (8)	
** Other**	23 (29)	12 (30)	11 (28)	
** Unknown**	5 (6)	2 (5)	3 (8)	
**Comorbidities, n (%)**				
** Hypertension**	50 (63)	25 (63)	25 (63)	0.99
** Diabetes Mellitus**	32 (40)	12 (20)	20 (50)	0.07
** Chronic Kidney Disease**	20 (25)	11 (28)	9 (23)	0.61
** Coronary Artery Disease**	11 (14)	4 (10)	7 (18)	0.33
** Cancer**	11 (14)	4 (10)	7 (18)	0.33
** Cirrhosis**	2 (3)	2 (5)	0 (0)	0.49
** Organ Transplant**	4 (5)	1 (3)	3 (8)	0.62
HIV	1 (1)	1 (3)	0 (0)	0.99

Characteristics of included individuals. iEpo “responder” is defined as an improvement in PaO_2_ / FiO_2_ of 10%.

At the time of iEpo administration, median PaO_2_/FiO_2_ was 92 (74 – 122). The ventilator mode for the majority of the patients (70 / 80, 88%) was assist control with volume cycling (AC/VC) and the others were on airway pressure release ventilation (APRV). Median FiO_2_ was 90 (70 – 100), and for the individuals on AC/VC, median PEEP was 12 cm H_2_0 (10 – 15) (Table 2).

**Table 2.** Clinical Features of iEpo Responders.

	Total (n = 80)	iEpo responder (n = 40)	iEpo non-responder (n = 40)	p-value
**SOFA score**, median (IQR)*	12 (11 – 15)	12 (10 – 14)	13 (11 – 15)	0.29
**Prone**, n (%)	46 (58)	28 (70)	18 (46)	0.02
**Fluid balance / day** mL, median (IQR)**	900 (120 – 2920)	860 (-320 – 2050)	1080 (370 – 3120)	0.29
**Respiratory parameters**, median (IQR)				
** FiO_2_**	90 (70 – 100)	100 (70 – 100)	80 (60 – 100)	0.19
** PaO_2_ / FiO_2_, mmHg**				
** Before**	92 (74 – 122)	86 (69 – 104)	102 (87 – 138)	<0.01
** After**	105 (81 – 145)	133 (98 – 171)	85 (64 – 109)	
** PaCO_2_**	45 (41 – 54)	45 (41 – 56)	47 (42 – 52)	0.98
**Ventilator parameters, median (IQR)**				
** PEEP, cm H_2_0**	12 (10 – 15)	12 (10 – 14)	12 (10 – 15)	0.34
** Tidal Volume, mL**	420 (360 – 490)	450 (360 – 490)	400 (360 – 500)	0.33
** Respiratory Rate**	22 (20 – 26)	20 (20 – 26)	22 (18 – 28)	0.43
** Plateau Pressure, cm H_2_0**	28 (25 – 30)	28 (26 – 30)	29 (25 – 31)	0.48
** Driving Pressure, cm H_2_0**	15 (13 – 19)	15 (13 – 17)	16 (14 – 19)	0.50
** Static Compliance, mL / cm H_2_0**	27 (22 – 33)	28 (22 – 34)	26 (22 – 33)	0.65
**Therapeutics, n (%)**				
** Neuromuscular Blockade**	12 (15)	3 (8)	9 (23)	0.11
** Anticoagulation**	14 (18)	5 (13)	9 (23)	0.37
** Steroids**	6 (8)	1 (3)	5 (13)	0.20
**Laboratory Data, median (IQR)**				
** C-reactive protein, mg/L**	169 (96 – 249)	162 (83 – 210)	171 (96 – 287)	0.27
** D-dimer, mg/L**	3.5 (1.3-8.5)	2.9 (1.6-8.3)	4.3 (2.1-8.9)	0.53
** IL-6 pg/mL**	43 (12-120)	55 (10-247)	42 (13-98)	0.63

* SOFA score calculated for the 24 hours around iEpo administration

** Fluid balance / day since intubation

### Change in Oxygenation

**Figure 1.**
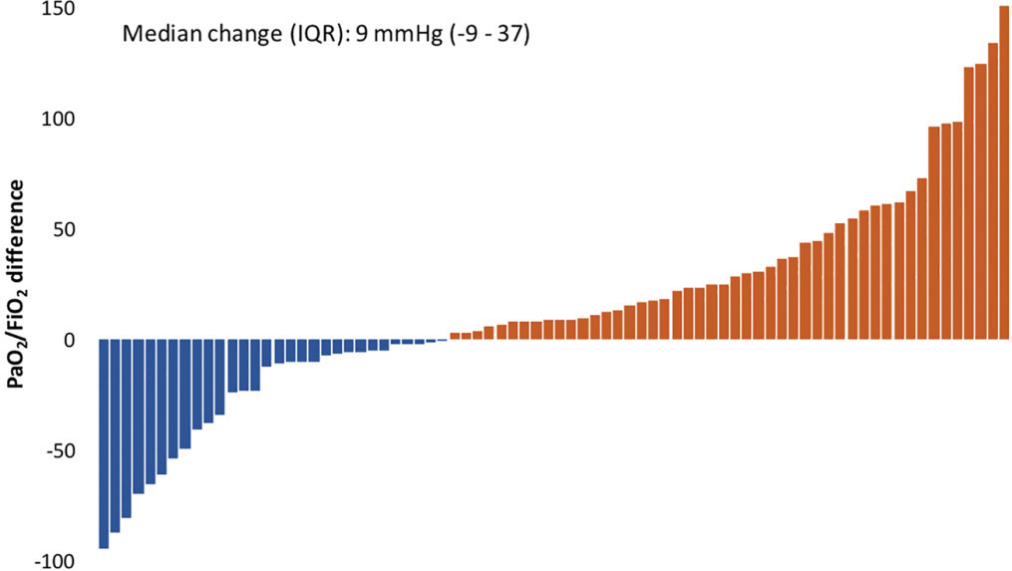
Change in PaO2/FiO2 after inhaled epoprostenol.

PaO_2_/FiO_2_ measurements were calculated from the arterial blood gases closest in time to the initiation of iEpo (before and after): a median of 2.9 hours (1.5 – 5.0) before iEpo was started and 2.9 hours (1.3 – 4.9) after. The median change in PaO_2_/FiO_2_ post-iEpo was 9 mmHg (-9 – 37) (Figure 1) corresponding to a 10% improvement (-8 – 41). Fifty-percent (40 / 80) met our a priori definition of a clinically significant improvement in PaO_2_/FiO_2_ (increase in 10% from the baseline value). The demographic and clinical characteristics of the “responders” and “non-responders” are described in Tables 1 and 2. There was no association between the time on iEpo and PaO_2_/FiO_2_. Static compliance did not vary between responders and non-responders (Table 2) and there was no linear association between static compliance and change in PaO_2_/FiO_2_ (p = 0.81).

The 2 variables associated with iEpo responsiveness were prone position and lower PaO_2_/FiO_2_ when iEpo was started (Table 2). For the 46 proned patients (56% of total), the median improvement in PaO_2_/FiO_2_ was 14 mmHg (-6 to 45) (vs. 3 mmHg [-11 – 20], p = 0.04 for supine individuals) corresponding to a 16% (-6 – 51) change from baseline (vs. 4% [-11 – 25], p = 0.05). Those with severe ARDS (as defined by PaO_2_/FiO_2_ < 100, n = 49) were more likely to have a 10% improvement in post-iEpo PaO_2_/FiO_2_ (59% vs. 35%, p = 0.03) with a median improvement of 16 mmHg (-2 – 46) corresponding to a 17% change (-2 – 59).

### Predicting Responsiveness

To further explore these univariate observations, we generated 2 multivariate regression models: a logistic model using a 10% improvement in PaO_2_/FiO_2_ as the outcome, and a linear model with continuous change in PaO_2_/FiO_2_ as the outcome. We included FiO_2_, PEEP level, time between measurements, the use of neuromuscular blockade and static compliance as additional covariates, despite the lack of statistically significant univariate associations, given their clinical relevancy to oxygenation. After this multivariate adjustment, lower PaO_2_/FiO_2_ at the time of iEpo initiation (adjusted OR 0.88 [95% CI 0.78 – 0.98] p = 0.04 for each 10 mmHg decrement in logistic model; coefficient -2.95 [-5.5 – -1.1] p = 0.03 in the linear model) and prone position (adjusted OR 2.5 [1.1 – 6.2] p = 0.05, coefficient 4.9 [0.9 – 7.8] p = 0.05) remain the only independent predictors of iEpo responsiveness in both cases. Of note, PaO_2_/FiO_2_ at the time of iEpo initiation did not vary among those who were prone vs. supine (92 mmHg [72 – 119] vs. 92 [79 – 143], p = 0.56); correspondingly, prone position was not more likely in those with severe ARDS (59% vs 54%, p = 0.70).

There was no difference in PaO_2_/FiO_2_ change based on ventilator mode. Median improvement was 7 mmHg (-9 – 27) for those on APRV and 9 (-9 – 43) for the remainder (p = 0.48). A subset of patients had marked changes (in both directions) after iEpo: 38% (n = 30) had a 25% improvement in PaO_2_/FiO_2_ over pre-iEpo values, and 16% (n = 13) had a 25% decrease. Individuals with a 25% improvement similarly had lower PaO_2_/FiO_2_ (85 mmHg [69 – 104] vs. 97 [84 – 125], p = 0.01) with a trend toward greater likelihood of prone positioning not meeting statistical significance (67% vs. 48%, p = 0.10).

## Discussion

The defining clinical characteristic of the SARS-COV2 epidemic has been respiratory failure, with profound hypoxemia leading to mechanical ventilation and overwhelmed intensive care units.^27^ While patients typically present meeting criteria for acute respiratory distress syndrome (ARDS), COVID-19 respiratory failure appears to be distinct in so far as it is steroid responsive,^28^ venous thromboembolism is common^29,30^ the course is protracted^31^ and there may be distinct phenotypes characterized by differing lung compliance.^17,32^ While clinicians have been advised to manage COVID-19 patients based on the existing paradigm for ARDS,^23^ these differences point to a different disease process, and the need to re-evaluate treatment strategies that are both established for ARDS and those that may be less frequently used as rescue therapies.^33^

An example is inhaled epoprostenol (iEpo), used as an adjunct therapy in the management of refractory hypoxemia. We identified 80 mechanically ventilated patients who received iEpo spanning 2 tertiary referral centers. Half had a clinically significant improvement in PaO_2_/FiO_2_ (≥10% over the baseline value); however, generally the benefit was modest (median improvement of 9 mmHg) with large variability (IQR -9 – 37), including a substantial number that had a marked responses in both directions. Prior studies of iEpo responsiveness in ARDS (before COVID-19) differ by dosing protocol, time between measurements, disease severity and lack uniformity in ventilator settings.^4–7,9,34^ Considering that, the median PaO_2_/FiO_2_ difference we observed is in the same direction but appears to be lower than previously published reports. It is notable that a significant worsening in oxygenation can occur. Perhaps in certain individuals iEpo aberrantly decouples the normal hypoxic vasoconstriction of pulmonary blood flow at a macro-level – but this is purely conjecture. If possible, clinicians should isolate the initiation of iEpo from other interventions to carefully assess its efficacy or lack thereof.

PaO_2_/FiO_2_ as an outcome has limitations. It fluctuates in the normal course of a patient’s illness^35^ and is dependent on FiO_2_, airway pressure and peripheral oxygen extraction.^36^ However, it is one of the primary measurements used by clinicians in the bedside care of mechanically ventilated patients, is correlated with mortality^37,38^ and is widely used as a factor in considering a patient’s eligibility for and response to therapeutic interventions. In the design of this study, we purposely excluded patients with FiO_2_ or PEEP changes between PaO_2_/FiO_2_ measurements to carefully examine the specific impact of iEpo. The timeframe after iEpo was administered before PaO_2_/FiO_2_ was checked was a median of 2.9 hours (1.3 – 4.9), after which a therapeutic response, if one exists, should be observed.^39^

The significance of the specific benefit for individuals who are prone or have a lower PaO_2_/FiO_2_ is not clear. It is possible that these observations are artefactual due to multiple comparisons; however, the associations remain significant after multivariate adjustment lending credence to the findings. Prior analyses of variables predicting outcomes with the use of iEpo either did not record prone position^4^ or excluded those individuals altogether.^7^ In the prone position, ventilation is more homogenously distributed throughout the lung whereas perfusion is relatively unaffected,^40^ generally improving ventilation-perfusion matching.^41^ This may lead to more balanced, efficient delivery of iEpo throughout the lung, thereby augmenting its efficacy. This is speculative, however, and the impact of this physiologic observation on patient outcomes is uncertain. That those with lower PaO_2_/FiO_2_ and severe ARDS (PaO_2_/FiO_2_ < 100) are more likely to have improved oxygenation and a robust response (>25% improvement) with iEpo may be informative for clinicians, but these are individuals in who iEpo would likely be trialed anyways. While the mechanism of this observation is not explained by the variables we collected, it is not driven by prone positioning (which was relatively equally distributed across PaO_2_/FiO_2_ strata). Notably, this is the opposite pattern to what one prior observational study previously described.^7^

This is the first analysis regarding the use of inhaled pulmonary vasodilators for COVID-19 that we are aware of. There are numerous limitations, however. It is retrospective, so measurements were made in the routine care of patients rather than accordance with a protocol. We attempted to mitigate the impact of a lack of a specific protocol by narrowing the cohort to those who did not have significant changes to their care during the study timeframe other than receipt of iEpo. Additionally, we did not track subsequent PaO_2_/FiO_2_ measurements while on iEpo. This was intentional: as time passes, the natural course of disease, ventilator manipulations, and other therapeutics (such as diuretics or steroids) likely play a large role in changes in oxygenation, limiting our ability to attribute differences to iEpo. Moreover, prior data suggests the benefit of iEpo may be lost over time.^34^ Gattinoni et al. popularized a theory differentiating phenotypes of COVID-19 on the basis of lung compliance,^17^ but we did not observe an impact of static compliance on iEpo responsiveness. However, other elements of these phenotypes, such as recruitability and specific imaging findings, were not explored. We did not address the role of iEpo in patients who are not mechanically ventilated or the impact on right ventricular afterload (given that invasive hemodynamic assessments were infrequently performed). Lastly, there was a relatively low rate of neuromuscular blockade – this was driven by recent data regarding is utility^15^ and drug shortages related to the pandemic.

## Conclusions

For individuals mechanically ventilated for COVID-19, we found evidence of early benefit to iEpo in 50% of patients, suggesting it may be useful as a rescue strategy. There is a large variability in responsiveness, however, including the potential for worsening oxygenation that clinicians should be aware of. Further research will be needed to establish any impact on long term outcomes.
